# Round the Hospitals

**Published:** 1920-07-10

**Authors:** 


### ROUND THE HOSPITALS.

The visit of the King and Queen to Edinburgh was extremely well-timed from a hospital point of view, for it has taken place in the midst of an enthusiastic and most carefully organised rally on behalf of the medical charities of the northern capital. The stimulus given by the added personal interest of their Majesties in the movement and their presence during the campaign for a quarter of a million pounds is incalculable. The King and Queen, with Princess Mary, devoted an hour on the afternoon of the 5th inst. to the Royal Infirmary, where the Royal party was received by Sheriff Crole, K.C., the superintendent, Sir Joseph Fayrer, the matron, Miss Gill, R.R.C., and others. The visit, as is customary with their Majesties, was regarded in the light of one paid privately to the patients, and only the surgeons and matron, with the sister of each ward, were present during the tour of the building. Two surgical wards and one medical were visited, and the sick were much gratified and cheered by kind and sympathetic words personally addressed to them. The nursing staff, with all patients able to be about, formed up to give a warm greeting to the King and Queen as they passed through the covered way leading from the surgical to the medical part of the building. Their Majesties, with Princess Mary, left a permanent record of their visit behind by signing their names, which will subsequently be carved into the wood, on a table prepared for this purpose.

---

Princess Alice, Countess of Athlone, presents prizes and certificates at a baby fête at Windsor in connection with the National Baby Week, as we go to press. This is one of the most crowded weeks of the season so far as efforts for voluntary hospitals and charities are concerned. On Monday there were matinées at London theatres in aid of the British Home for Incurables, Streatham, and the Metropolitan Ear and Throat Hospital, besides the American Syncopated Orchestra performance for the National Institute for the Blind. On Thursday Princess Beatrice opens a sale of work and garden-party in the grounds of Mount Vernon Hospital, Northwood; Prince and Princess Arthur of Connaught open a garden fête at Chiswick House in aid of the West London Hospital; Princess Alice, Countess of Athlone, opens the Princess Alice Nurses' Home of the Middlesex Hospital, and will be present at the garden-party; the Duchess of Somerset opens a bazaar in aid of the East London Nursing Society at 77 South Audley

**ROUND THE HOSPITALS—(*continued*).**

Street, and the Dowager Countess of Airlie will open a sale of work in the Twining Ward of King's College Hospital, where a garden fête will be held. On Friday Lady Irene Curzon will open the fête organised by the students at the Royal Free Hospital, to which the Queen has sent a picture.

The King held an Investiture on July 5 in the forecourt of Holyrood Palace, where a red and white pavilion was set up under the shadow of Queen Mary's Tower, and the following members of the Nursing services received decorations from His Majesty's hands on this historic occasion:—*Royal Red Cross, Bar*: Miss Janet Melrose, T.F.N.S.; *First Class*: Miss Catherine Roy, Q.A.I.M.N.S., also received the Military Medal; Miss Elizabeth Cumming, A.N.S.R., and Miss Helen Fraser, C.N.S.; *Second Class*: Miss Dorothy Scott-Erskine, Q.A.I.M.N.S., Miss Annie Nicoll, Miss Jean Roy, and Mrs. Christabel Thomson, Q.A.I.M.N.S.R.; Miss Alexina Cameron, Miss Catherine Matheson and Miss Margaret Walker, T.F.N.S.; Miss Isabella Dodds, Miss Mary Duncan, Mrs. Mary Elliott, Miss Mary Grassick, Miss Hannah Glendenning, Miss Jane Gregorson, Miss Alice Macdonald, Miss Johan McKinnon, Miss Jessie McLean and Miss Annie Paterson, B.R.C.S.; Miss Margaret Montgomery and Miss Jean Wilson, V.A.D.

The "Sophy Hall" Surgical Ward at the Elizabeth Garrett Anderson Hospital was named on July 5 in honour of Lady Hall, the Chairman of the Appeal Committee and originator of the Fund. The ceremony consisted in drawing back the curtain from the ward window, when the Coat-of-Arms of Colonel Sir John Hall appeared in stained glass in the centre light, with two scrolls, "Sophy Hall inspired and led the memorial appeal" and "The heart to conceive, the understanding to direct," on either side. The Chairman of the hospital, Mr. Archibald Gordon Pollock, sent a great basket of roses, and a bunch was laid on every surgical bed. Sir William Plender gave a brief account of the fund raised, on Lady Hall's initiative, to commemorate the fiftieth anniversary of the hospital. The sum asked for was £50,000, and it is now all in hand or promised. Lady Hall has presented a bronze statuette of Florence Nightingale to the ward named after her.

The report of Queen Victoria's Jubilee Institute for Nurses for 1919 is one of wide national importance. The business of bringing trained nursing into the home of every member of the community is now in a fair way to be accomplished. No longer patchy or fragmentary, this organisation has, with the aid of twenty-nine County Nursing Associations, covered nearly the whole of the British Isles with a network of nursing societies which become every year more stable and better co-ordinated. In spite of the scarcity of nurses, the total of Queen's nurses working on January 1, 1920, amounted to 1,954, and the total number placed on the roll during the year was 121, as compared with 101 the previous year. The number of resignations is too high at 324, but as a set-off seventy former Queen's nurses rejoined the Institute. Financially the Institute has benefited largely from the Nation's Fund for Nurses, which has allocated £7,000 to assist its general purposes, and the Queen's Nurses' Benefit Fund in England and Scotland. The need for an increased income is very urgent. Training and inspection are both far more costly than they used to be, and many districts are calling in vain for Queen's nurses.

A highly interesting feature of the year's work of Q.V.J.I.N. is the permission given to two Queen's nurses to hold their badges and brassards while working as district nurses in France, and to a third who is undertaking district work in Italy. The districts would be affiliated in the ordinary way were it possible to make any satisfactory arrangement under which the work of the nurses could be inspected. But these Queen's nurses are acting as pioneers in a kind of nursing hitherto altogether unknown in France and Italy, and there is great hope that this may lead to the initiation of organised systems of district nursing in both countries. District nursing in England started some sixty years ago with one nurse in Liverpool, on the inspiration of Mr. William Rathbone. Another sixty years may see the same system covering Europe.

The extraordinary difficulties now existing in the building world are shown in the experience of the Bermondsey Board of Guardians, who are desirous of extending their nurses' home. Their architect prepared the necessary plans, and estimated that the work would cost £16,000; but the lowest tender received was for £34,000. The Ministry of Health, whose sanction to proceed with the work was required, have at present declined to give it in view of the extraordinarily high cost, and have suggested to the Guardians that they might utilise the casual wards near their infirmary, and convert them into accommodation for the nursing staff. But the casual wards have not been used for some years, and face the mortuary—not at all a desirable site for a nurses' home.

Lady Astor does well to remind the public, as she has lately done, that there are still nearly 10,000 Service and ex-Service men in hospitals in or near London and to urge upon all who can do so to share in their degree in the duty of helping to cheer them. She has herself lately had one or two parties of soldiers, still in hospital blue, at the House of Commons, and has realised from talking to them that outings and entertainments are no longer being given on the same generous scale as during the war. When it is remembered that some of these men have been three or four years, or even longer, in hospital, the case for enlivening their existence becomes very strong indeed. Lady Astor suggests river trips, parties in private gardens, motor drives, and money

**ROUND THE HOSPITALS**—(*continued*).

to provide chars-à-banc drives. She specifies the patients at the Maudsley Hospital for nerve cases at Denmark Hill as particularly suitable for such attentions, and quotes Pastor John Stanley, 29 Gloucester Street, S.W. 1, as secretary of a society doing good service in arranging outings for wounded sailors and soldiers.

---

The inquiry into the management of the Newark General Hospital has ended and Mr. Frederick Acton, C.B.E., who conducted it has reserved his report. The most important witness was Dr. E. Ringrose, who attended the complainant, Mr. Ransome, during his term of residence in the hospital. He gave it as his opinion that the nursing staff was over-worked, the management having economised at the expense of efficiency in regard to its nursing staff. Some of his patients in both male and female wards (though not Mr. Ransome) were dirty, and a good number were suffering from sore backs; he sometimes found the wards unattended and analyses were not taken. He did not think there were enough trained nurses, and in his judgment some of the beds ought to have been closed. His complaints, however, were always attended to. Except for a few general complaints he was not dissatisfied with Mr. Ransome's treatment while in hospital. A considerable amount of testimony was given by members of the nursing staff and the honorary medical officers to the excellence of the hospital management and the integrity and exemplary character of the matron.

---

The Hampstead Guardians have decided to allow their resident probationers a messing allowance of 15s. a week each, instead of a fixed dietary with food supplied by the Guardians. The staff are to appoint a mess committee with president, and the experiment will be watched with great interest, and we trust, if successful, command imitation. They are suffering from an acute shortage of nurses at the infirmary, but an advertisement for probationers desirous of "living out" produced only one applicant, so evidently that is not the remedy. Probationers' salaries at Hampstead run from £30 to £38.

---

We have received a copy of a letter, signed J. P. Lucas, which has been addressed to the Minister of Health in view of the probability that the London and other hospitals may be shortly in receipt of public aid. The writer urges that if public funds are to be used for the assistance of voluntary institutions it should be made obligatory to improve forthwith the lot of the nurses and probationers on whose work the hospitals depend. He alludes to the "intolerable hours" imposed on nurses and to Dr. Addison's own statement that their pay is that of a scullery-maid, and sums up a strong case by declaring that "with reasonable hours and improved pay the complaints of the scarcity of recruits to the nursing ranks would disappear." We are inclined, however, to dispute the writer's contention that it is the sweating of nurses by excessive working hours and inadequate pay which is responsible for the fact that only some ten per cent. of the people contribute to the support of these institutions.

---

The Plymouth Poor-Law Infirmary appears to be deplorably under-staffed. One of the sisters has recently resigned "on account of too much responsibility," and a lady guardian, commenting on this resignation, testified to the fact that there were sixteen children suffering from whooping-cough who had been left without a nurse all the afternoon. Many of the patients, she declared, were suffering from lack of nursing.

---

It is difficult to understand the anxiety expressed by the Central Midwives Board lest midwives should gain too much power on the Board. In the new constitution proposed it is stipulated that the number of midwives on the Board shall at no time exceed five. There will be fourteen persons altogether. Of these two out of the four appointed by the Minister of Health must be midwives, while two more midwives are to be appointed by the Incorporated Midwives' Institute. It is extremely likely that Queen Victoria's Jubilee Institute for Nurses will choose a midwife for their representative. Four registered medical practitioners make up the prescribed number as far as eleven. The remaining three persons are to be appointed by the Association of County Councils, the Association of Municipal Corporations, and the Society of Medical Officers of Health. None of these bodies is to be at liberty to choose a midwife unless the Q.V.J.I. has omitted to do so. The professional training which admits to the Midwives' Roll may be only one among a number of other qualifications possessed by a possible representative, but it is considered of a nature to disqualify its possessor from assisting at the deliberations of the Midwives Board if she be reinforced by as many as five others in a similar plight. We are not sure that this is not a compliment in disguise to the forceful qualities of the midwife.

---

Miss Cann, the Matron of Batley and District Hospital, Yorks (fifty beds), has lately retired, after twenty-five years' service, and a remarkable tribute of respect and gratitude was offered to her at the farewell meeting, when the President of the hospital, the Mayor and Mayoress, and a large and representative company assembled on the hospital lawn to express their thanks. The hospital has granted Miss Cann a pension of £52 a year. The Mayor on this occasion presented her with an illuminated address and a cheque for £500. The ex-patients have subscribed to provide her with a handsome travelling-bag, having shown their love for the Matron and her work by raising about £1,200 during the last ten years for the hospital. Many hearty expressions of admiration came from those present who had witnessed and shared Miss Cann's labours for the sick. A striking testimony was

**ROUND THE HOSPITALS—(*continued*).**

afforded by a life governor of the hospital who was once a patient: "I have passed through the hands of about 150 nurses," he declared, "but truthfully I have never found one so skilful and clever as Miss Cann. Hers was a healing personality."

The march of the boys from the Robert Montefiore School, E.C., past the London Hospital, where they all dropped pennies into the milk churn to help to pay the milk bill for the patients, was a pleasant sight, and was a real object-lesson in the art of making it interesting to give. The girls and infants are planning a march on their own account, and if the idea will only catch on we may have processions of all kinds arranged for the same purpose. Every child enjoys the art of giving when the gift is bestowed personally, with a little ceremony, and for a purpose familiar to the giver. The hint is worth taking, and is capable of wide application. A march past of donors might become a very popular feature in hospital fêtes. But apart from this, every act is wholesome which impresses on the young the duty of caring for the sick poor.

Considerable interest attaches to the appeal lodged at Bow Street Police Court on July 5 by Mrs. Florence Maud Johnson, against an order of the London County Council cancelling the registration of her as a person carrying on a lying-in home at Lewisham. The notice which Mrs. Johnson, whose home was used for "twilight sleep," received that her registration was about to be cancelled stated two reasons: (*a*) "That the Council has reason to believe you are of bad character"; (*b*) "That the premises and equipment are unsuitable for the purposes of a lying-in home." Evidence of unsuitable equipment was to the effect that silk instead of catgut had been used on one occasion to sew up a wound, and that once newspaper had been used instead of waterproof sheeting. As regards character, Mrs. Johnson stated that the man Tattersall, whose presence in the home in a room leading out of hers had been objected to, was the adopted son of herself and her late husband. The hearing has been adjourned.

Many of the smaller hospitals in the country are increasing their medical and nursing staffs, and in view of the scheme propounded by the Consultative Medical Council to the Ministry of Health it is highly probable that there will be a great development of work in such centres. At the Lowestoft and North Suffolk Hospital, where the number of beds is forty, the matron has hitherto acted as theatre sister. This undesirable arrangement is now to come to an end, and the staff will be enlarged to admit of a sister undertaking this exacting branch of work. There are to be five senior and five junior surgeons, instead of three as at present, and various other administrative changes will take place. Lowestoft residents undertook that a new and up-to-date hospital should be one of their thankofferings for victory and memorials for the fallen, but, like some other places with the like aspiration, they have failed to realise the amount of hard cash needed to carry through such a project.

An influential meeting, under the presidency of the Lady Mayoress, Mrs. W. Cadbury, was held at the Council House, Birmingham, on June 21, under the auspices of the College of Nursing, Birmingham and Three Counties Centre. Its object was to explain a scheme and attract support for a scheme to further the interests of nurses. The plan proposed is to form a club and hostel for trained nurses, with lecture hall and restaurant, and a scenic fair is to be held next spring with this aim in view. Miss Musson explained to the meeting the disadvantages under which nurses now labour and the small inducements offered to educated women in the profession. Sir Gilbert Barling cordially supported the scheme, and believes the public is more interested in the general welfare of nurses than in raising their educational standard.

The shortage of candidates is quite as acute in South Africa as in England. At the annual meeting of the South African Trained Nurses' Association it was proposed "that it is advisable that the age at which probationers may be admitted to hospitals for training be lowered to a minimum of nineteen years." There was a good deal of opposition, and it was stated that the Cape Provincial Council had rejected this proposition, but the motion finally passed by fourteen to thirteen votes. On the question of a registered uniform for members of the Association much divergence of opinion became evident, but it was finally agreed that a badge woven into the material of which the caps were made should be registered as the property of the Association.

The Durham County Nursing Association, one of the latest in the field, held its second annual meeting on June 18 in circumstances of great encouragement. There are now thirty-five affiliated nursing associations, a gain of fifteen in the year, and several others forming. There is at present no accommodation in the county for lying-in mothers and none for training midwives, but the County Council has acquired, and will shortly equip, premises suitable for these purposes at Clairmont, Bishop Auckland, and one other centre. This should do much to wipe out the existing shortage of qualified midwives for service in the county.

At the Glasgow Hospital for Sick Children we learn from the annual report that the nursing staff is now working generally on a basis of fifty-six hours per week, a considerable curtailment of the former hours on duty, entailing the addition of more probationers. There were 250 applicants for thirty-four vacancies, which is really not bad.

